# An exploratory qualitative study of the psychological effects of HIV diagnosis; the need for early involvement of mental health professionals to improve linkage to care

**DOI:** 10.1186/s12889-023-17449-y

**Published:** 2023-12-15

**Authors:** Jerry Paul Ninnoni, Frederick Nsatimba, Sampson Opoku Agyemang, Isaac Tetteh Commey, Lydia Bennin, Elizabeth Agyare, Leveana Gyimah, Kafui Senya, Nyonuku Akosua Baddoo, Dorcas Obiri-Yeboah

**Affiliations:** 1 Department of Mental Health, School of Nursing and Midwifery, University of Cape Coast, Cape Coast, Ghana; 2 Public Health Unit, Cape Coast Teaching Hospital, Cape Coast, Ghana; 3 Communicable and Non-Communicable Diseases cluster, World Health Organisation Country Office, Accra, Ghana; 4 National AIDS/STIs Control Programme, Accra, Ghana; 5 Department of Community Health, the University of Ghana Medical School, Accra, Ghana; 6 Department of Microbiology and Immunology, School of Medical Sciences, University of Cape Coast, Cape Coast, Ghana

**Keywords:** HIV, Diagnosis, Interpretation, Psychological, Ghana

## Abstract

**Background:**

Diagnosing a life-threatening disease like the human immunodeficiency virus (HIV) can be unbearable to the individual, which has implications for their subsequent care-seeking decision-making. However, an essential element of HIV testing is identifying infected individuals and linking them with adequate care services, thus contributing to the UNAIDS 95-95-95 targets. The attainment of these targets has been particularly challenging for lower and middle-income countries (LMIC). This study explored the psychological reactions to a positive HIV status in a hospital treatment centre to provide insight into mental health interventions’ role in improving HIV screening and early antiretroviral therapy (ART) initiation to enhance the quality of life.

**Methods:**

An exploratory qualitative study was investigated among adults who were diagnosed as HIV positive. Participants were purposively recruited from an HIV Treatment Centre. Data were collected with semi-structured interviews that explored the interpretations and psychological reactions to their positive HIV status. Overall, 18 participants were interviewed to reach saturation. Data were transcribed verbatim and analysed thematically to produce findings that address the study’s objective.

**Results:**

Following analysis of participants’ interpretations, understanding and implications of their HIV-positive diagnosis, two major themes emerged: (1) anxiety regarding the impact of the disease on self, family and society was overwhelming. Participants were anxious because of the stigma, fear, worry, shock, and shame they faced. (2) Participants expressed hopelessness and could not see meaning or purpose in life. Suicidal ideation, suicide plans and self-harm characterised hopelessness.

**Conclusions:**

The initial reaction to the diagnosis of HIV in this LMIC context has the potential to impact linkage to care negatively and, thus, the attainment of the global 95-95-95 targets. It is, therefore, essential that mental health and psychological support services are integrated with testing services to manage the initial reactions and support individuals to improve early linkage to care and thus improve overall outcomes for the infected individual and society.

## Background

Diagnosis with a chronic illness, including human immunodeficiency virus (HIV) infection, may trigger adverse reactions among individuals depending on their interpretation of the disease. These psychological effects may lead to poor linkage to care and services if not promptly and sufficiently addressed. The population of people with HIV in Ghana is estimated at 361 797, and new HIV infections at 11,705. Adult [1–35] prevalence is an average of 1.7% (2020–2022), although the prevalence is higher among other high-risk populations, such as female sex workers and men who have sex with men [36]. The annual HIV-related deaths total 5,312 [37].

UNAIDS launched an ambitious target of 95% of all individuals with HIV to know their HIV status, 95% of all who are diagnosed with HIV to receive sustained antiretroviral treatment and 95% of all who are on antiretroviral therapy (ART) from having viral suppression by the year 2030 known as the programme 95-95-95. Following this, Ghana records 71%, 99% and 79% [37]. This suggests that 71% of all individuals with HIV in Ghana know their HIV status, 99% of those diagnosed with HIV receive sustained antiretroviral treatment, and 79% are on antiretroviral therapy to have viral suppression [37]. Ghana has achieved significant progress in the fight against HIV/AIDS. For example, Prevention of Mother-Child-Transmission (PMTCT) coverage is estimated at 100%, and ART coverage for all adults is estimated at 84% [37]. The National AIDS Control Programme offers three avenues for HIV Testing and Counselling (HTC) in Ghana, including facility-based Diagnostic Centres; outreach Centres known as the Know Your Status Campaign (KYC) and training through PMTCT. PMTCT services are available at all public and private health centres in Ghana and reflect the WHO strategy for the prevention of HIV in mothers of reproductive age, the prevention of unintended pregnancies among people living with HIV, the prevention of HIV from women living with HIV to their children and the provision of appropriate treatment care and support to mothers living with HIV and their Children [38]. According to the Guidelines, all pregnant women are offered HTC services at registration. However, they have the right to refuse testing.

Despite these efforts to fight HIV/AIDS, roughly only 71% of people are aware of their HIV status [37]. The uptake of HIV testing services remains low in the country, particularly among high-risk ones, leading to a high rate of undiagnosed cases [37]. A recent study among the youth (15-24-year-old) observed that all participants were knowledgeable about HID/AIDS, including the mode of transmission, and 80.5% were aware of the Know Your HIV Status Campaign (KYC) and the Voluntary Counselling and Testing (VCT), less than half tested for HIV and a few utilise the VCT services [39]. It is claimed that the fear of testing positive and the associated stigma discourage people from getting tested for HIV and serostatus [39].

Furthermore, it is claimed that conventional facility-based and provider-assisted HIV Testing Services (HTS) have some barriers to universal access to testing and treatment. These barriers include stigma, negative provider attitudes, discrimination, limited confidentiality, and limited convenience [40]. The potential of HIV self-testing (HIVST) to increase HIV testing uptake as an entry point to the HIV/AIDS care continuum, especially among the highly stigmatised and hard-to-reach populations, is well-documented [1–3, 41–46]. It is reported that convenience, confidentiality, and privacy are highly influential in the acceptability and utilisation of HIVST [40, 45]. HIV testing, whether self-testing or referred, is essential for many reasons. For example, acceptance of the HIV-positive status is essential for engagement in treatment regimes, social networks, transmission risks, stigma reduction and disclosure activities [45]. Also, difficulties accepting the diagnosis may lead to health and psychological consequences for the individual and the general public, including mental health conditions such as depression and Posttraumatic Stress Disorder (PTSD). Following the initial diagnosis of HIV, patients are reported to experience several catastrophic and distressful psychological reactions [47]. A positive diagnosis is often associated with a sense of loss, purpose and hope [41]. A similar study reported clients voicing suicidal ideations following a positive diagnosis [48], and another study found that one-fifth of the people experienced suicidal ideation within a week after a positive diagnosis [49]. This also suggests that trends of suicide and suicidal ideations may vary in the course of the HIV diagnosis. It is claimed that the first six months are potentially high risks for suicidal ideation [42] and that the riskiest periods are within the first three months of diagnosis [43]. Evidence suggests that up to 40% of HIV-positive individuals are at risk of suicide within these three months [44, 45]. These thoughts often reflect the perception that HIV has no cure despite advances in the pharmacotherapy [46]. Furthermore, a study in Sub-Sahara Africa reported that 55.3% of people living with HIV with Posttraumatic Stress Disorder (PTSD) and another 41% of respondents met the criteria for a probable diagnosis of PSTD [1]. Individuals with a possible diagnosis of PTSD were associated with having experienced an adverse life event in the past six months [1].

Stigma and some sociocultural perspectives influences including HIV-positive testing [46]. In particular, religion and religiosity are reported to be protective and risk factors for spreading HIV [2]. Adherence to religious teaching and principles has been thought to protect against HIV/AIDS transmission [3]. Society’s attitude toward HIV is perceived to be related to its deviation from ethical norms and practices. As a result, participants reported they kept their diagnosis secret [49]. They conceal their condition from people because they are uncomfortable disclosing their status to others. The psychosocial well-being of individuals with HIV can be affected by HIV-related stigma through direct experiences of prejudice and discrimination and the anticipation of being stigmatised or the fear of being discriminated against [4, 5]. These may influence the individual reaction to the disease and their health-seeking behaviours. Identifying the individuals’ reactions to a new diagnosis of HIV-positive may facilitate access and retention of those individuals in the health system for specialised interventions. Although there are numerous studies on HIV, including HIV-related stigma in Sub-Sahara Africa, a dearth of research focused on investigating the psychological reactions to the initial diagnosis of HIV in Ghana. This study explored the interpretation and psychological responses to a positive diagnosis of HIV in a referral centre in Ghana.

## Methods

### Design and setting

An exploratory qualitative study was conducted at the Cape Coast Teaching Hospital (CCTH) in Ghana between May and December 2021. This hospital is one of the biggest referral hospitals in Ghana and has the largest Antiretroviral Therapy (ART) Treatment Centres in Central Ghana [6, 7]. The hospital is a 400-bed capacity. It is affiliated with the Medical School, School of Nursing and other Health and Allied Sciences programmes.

### Population and sampling techniques

The study population included all adults aged 18–72 years with a confirmed HIV status and registered with the hospital for treatment for at least a year.

### Data collection

Data were collected using in-depth face-to-face interviews by one of the ten research team members with experience in qualitative interviewing. All participants were allowed to choose and assign pseudo names. The interviews were conducted at designated offices at the treatment clinic within the hospital. The time for each discussion was agreed upon with the participant. Interviews lasted an average of 30 to 35 min. The questions explored HIV diagnoses, interpretation and reactions. Interviews were analysed until saturation was reached and confirmed by an additional two interviews totalling 18 participants. The interviews were performed in the patient’s local language of choice and translated into English by the interviewer, who is proficient in both languages using Brislin’s approach [1]. A procedure where the interviewer, fluent in both English and Fante (the local language), translates the local language into English. An equally proficient translator then reviews the translated versions with the originals, adjusting as necessary to ensure consistency and agreement [8]. This approach preserves the transcripts in the original voice of the participant during coding so that the transcript can maintain its linguistic complexity and cultural context [9]. In addition, all researchers kept field memos in which personal reflections and biases were recorded to support the interpretation of the data.

### Data analysis

The data were analysed thematically. First, each interview was transcribed verbatim, and the transcripts were checked for consistency and accuracy. Next, the transcripts were read several times to enable familiarisation with the data to appreciate the text and context [10]. Initial codes were generated following familiarisation, having identified ideas about what was interesting about them [11, 12]. Next, the transcribed data were coded. All relevant coded extracts were sorted into themes relating to the individuals psychological reactions and effects of diagnosis to HIV [11]. Next, team members independently reviewed the emerging themes to identify patterns for the validation [11, 13]. Afterwards, the research team met as soon as the coding was completed to confirm the emerging themes and sub-themes participants described. This lasted for about two hours. Several themes and sub-themes were identified as areas of similarities, overlaps within and between codes, and re-categorisation for fitness. The final themes that emerged were then compared to the research objectives. Finally, findings are reported using direct participant quotations to validate the phenomena.

### Ensuring trustworthiness

Trustworthiness was ensured [14]. This involved credibility, transferability, dependability, confirmability and audit trail [15]. Credibility represents the ‘fit’ between respondents’ views and the researcher’s representation of them [16]. This was facilitated by prolonged engagement [13]. Detailed descriptions of the study design have been provided to inform transferability in a similar context [16]. This also allows the study process to be examined to judge the study’s dependability [7]. Trustworthiness was also ensured by checking the findings to ensure the transcripts were accurate and by assessing the consistency of themes by research team members [15]. Two team members reviewed the transcripts and assigned codes to various categories of data. All identified codes had relevant implications for the research objective. The participants validated the findings through member checking [17]. Finally, an audit trail of all activities during the study was kept. This is demonstrated by keeping a record of how the study was carried out and how researchers arrived at conclusions. A description of all the steps taken throughout the study, supported by a comprehensive collection of relevant data, including field notes and memos [18].

### Ethics approval

The study followed the Helsinki Declaration and was approved by the Hospital Ethics Committee (CCTHERC/EC/2021/028). Ethical principles such as voluntary participation and confidentiality of the information were explained to respondents to obtain informed consent was sought. All COVID-19 protocols were considered.

### Demographics of participants

Table 1 is referred to. Most participants were women [45], and few were still married [40]. In addition, most were at least 40 years older [46], and most had the disease for at least five years [46].

**Table 1:** Participants’ Demographic Information (N = 18)

No.	Pseudonym	Gender	Age (yrs)	Marital status	Religion	Education level	HIV Duration (in years)
1.	Adwoa	Female	48	Divorced	Christian	Primary	4
2.	Kwame	Male	45	Divorced	Christian	SHS	6
3.	Joyce	Female	40	Divorced	Christian	JHS	9
4.	Ama	Female	67	Widowed	Christian	JHS	5
5.	Rita	Female	31	Cohabiting	Christian	SHS	7
6.	Serwaa	Female	42	Cohabiting	Muslim	JHS	10
7.	Yaa	Female	38	Divorced	Christian	Vocational	8
8.	Lizzy	Female	46	Married	Christian	None	12
9.	Daniel	Male	72	Married	Christian	JHS	5
10.	Emmanuel	Male	69	Married	Christian	JHS	7
11.	Elorm	Female	55	Widowed	Christian	None	6
12.	Kofi	Male	52	Divorced	Christian	JHS	3
13.	Sandra	Female	47	Single	Christian	None	13
14.	Akosua	Female	40	Married	Christian	SHS	4
15.	Esther	Female	56	Divorced	Christian	JHS	9
16.	Joseph	Male	70	Divorced	Christian	JHS	11
17.	Millicent	Female	38	Married	Christian	JHS	5
18.	Esi	Female	33	Single	Christian	Tertiary	3

**Fig. 1:**
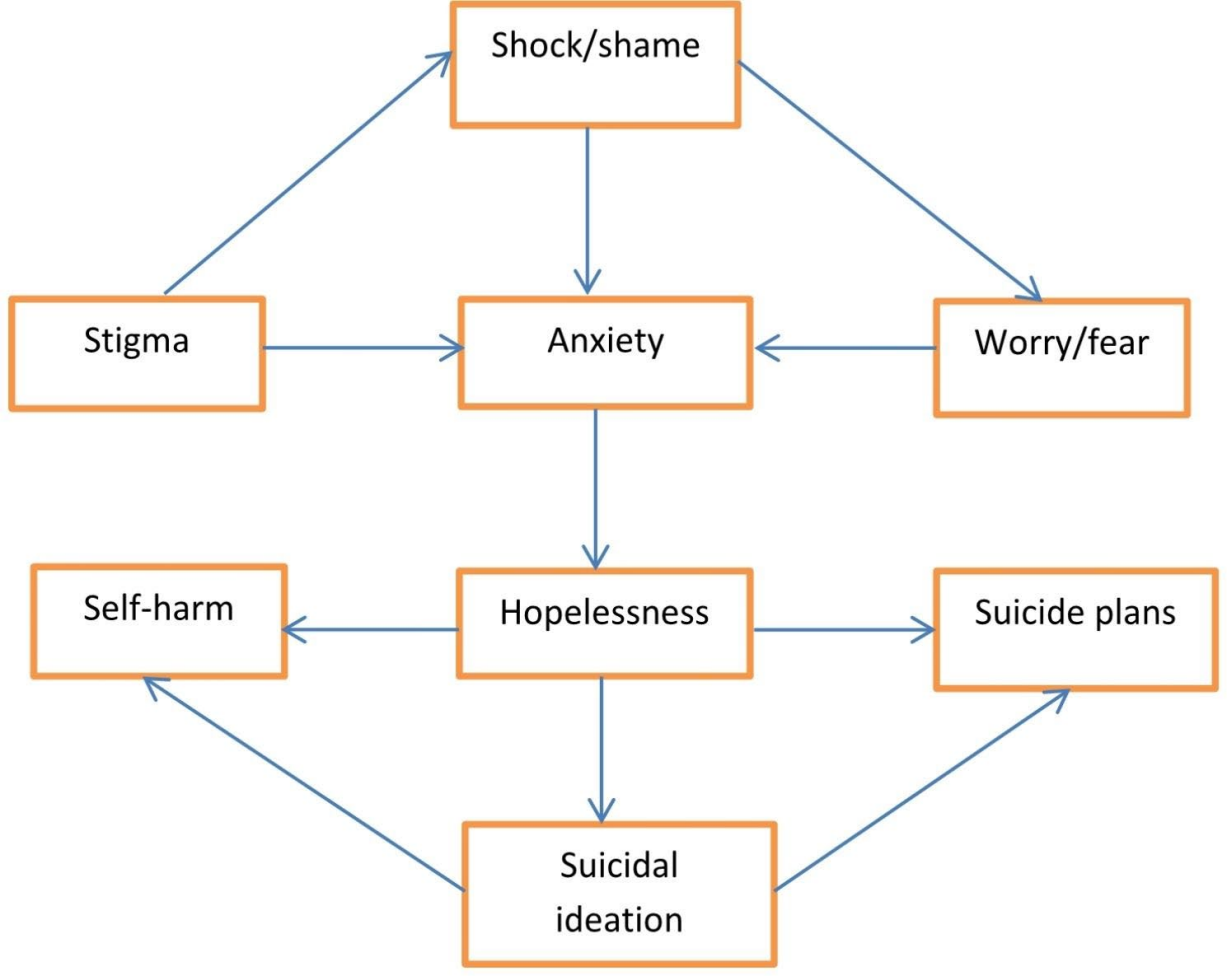
Key findings: Themes and Sub-themes of psychological reactions

## Results

Data analysis on psychological effects HIV-positive diagnosis resulted in two main themes and six sub-themes. (1) Participants reported the anxiety caused by the diagnosis and how it impacts self, families and society. Subthemes include; stigma, shock/shame, fear and worry. (2) Participants also reported hopelessness upon discovering they were HIV- positive. Subthemes include; suicidal ideation, self-harm and suicidal plans, as shown in Fig. 1 above.

### Hopelessness

Participants reported despair and loss of hope upon hearing they were HIV-positive. As a result, participants contemplated suicide and voiced suicidal ideations.

### Suicidal ideation

Participants contemplated suicide upon discovering they were HIV positive.

“When *I was told I had this disease, I almost poisoned myself … I locked myself in the room, and all I could do was cry”*, Serwaa.

*“I became scared and was even tired. Finally, I said God should let me go. I used to complain to my elder sister in the US. She said I shouldn’t do that,”* Kwame.

### Self-harm

Other participants resorted to self-harm when they discovered their HIV-positive status.

“*In the beginning, it was difficult for me; I wasn’t even eating because what was the point eating* ”, Adjoa.

“*It was difficult for me in the beginnin*g *and I started drinking alcohol* ” Serwaa.

### Suicidal plans

One participant disclosed that the diagnosis of HIV infection pushed him to prepare an active suicide plan to share his properties with his children:

“*In the beginning, when I was told, truly I went crazy, so I made up my mind if so, then I will call my children and share the little I have for them because I was out of time and wanted to die”* Daniel.

### Anxiety

Anxiety was reported as a critical theme by participants. Most participants were anxious regarding the impact of the diagnosis on self, families and society’s perception of them. Three sub-themes were reported: Stigma, shock/shame and worry/fear.

### Self-stigma

Participants reported self-stigma and the public after being told they were HIV-positive.

*“Yes, you see what this disease looks like… people think that if you involve yourself in prostitution, that is how you get it, so I was wondering what people will think about me when they learn about it”*, Serwaa.

*“ No, I haven’t done that before; apart from my children, who else can I even mention*.

*this to apart from the doctors taking care of me? Even though I haven’t told them if Someone suspects or knows about it, the person hasn’t said anything to me. I don’t think it will help me in any way if I tell someone because the person may end up gossiping around,”* Adjoa.

*“ If you go to the hospital and they ask if you have any disease, how do you mention it?*

*It makes you less of a person; people will even think you did something bad that made you get it,”* Ama.

*“No!…. even when people suspect you, they avoid you; how much more you tell them yourself, they won’t even come close to you at all so that they will even comfort you”* Serwaa.

*“ I can’t tell anyone, and I am not willing to do that because this disease is embarrassing,”* Lizzy said.

*“I have not told anyone, as I said before, apart from my mother and siblings and my two uncles nobody knows, even my friends, they don’t know. But, because I have this thing, I am not too public like I used to be before. So I stop outings, even the beach. I stopped going because I must be cautious,”* Kwame said.

### Stigma

Other participants also reported public or anticipated stigma

*“When I heard about the disease, because of how people described it, I got terrified, so I wept throughout”* Akosua.

*“Hmm, it is tough for me to tell people because you don’t trust people. If you think telling someone it won’t cause any problem, that person may be broadcasting it. So apart from the hospital, that they may ask me, and someone gets to know about it, I can’t tell someone about it. When people get to know you, they will see you as unclean. Some will even insult and embarrass you, but I can’t,”* Ama.

*“Mmm… yes, because most people think that it is when you engage in sexual activities that you get it, so if they see you have it, they say many bad things behind you… they may even avoid you; you can’t even buy food or something… they won’t sell to you… they keep pointing fingers anytime you pass by” Rita*.

*“Ehm… I wish they had changed how they speak to us; if not for the disease, some older adults wouldn’t have come here. But, unfortunately, the stigma and discrimination will not even make people come here,” Rita.*

### Fear/worry

Participants reported a wide range of concerns of great worry to them following discovering they were HIV-positive:

Some participants indicated they were afraid and worried because of their dependents and children. In the event of their death, a source of worry is how their dependents will be cared for.

*“In the beginning, I worried that my children were still young, and I was afraid I would die, leaving them to their fate, so it got me thinking a lot*”, Adjoa.

*“There are several worries; for instance, since my husband died, I am the only person catering for my children. Upon all these, I have also been operated on one of my breasts. I can’t marry again; at least the person would support me financially*,” Elorm.

Other participants were worried about education, marriage and childbearing and feared they might be unable to marry and have children.

*“Yes,… particularly about my marital issues and the desire to further my education and the profession of my choice… I feel I may miss all of these. Even childbearing because if you meet a man and tell him this is your problem, the man will walk away. And about childbearing too, I already have one child by then. I didn’t know I had this disease, but because of the disease, if I meet a man and get pregnant, I am left with no option but to abort,” Rita said.*

Some participants became sad and wept when they learned they were HIV positive.

*“The fact was when I got to know I have this kind of disease, I became very sad,” Rita said.*

*“I worried my children were still young, and I was afraid I would die, leaving them to their fate, so it got me thinking a lot”*, Adjoa said.

Some were worried because they had other health conditions.

*“Yes, I am worried because I have diabetes already and now this one. I don’t worry about diabetes, but this very disease… if you go to the hospital and they ask if you have any disease, how do you mention it? It makes you less of a person; people will even think you did something bad that made you get it. So when they told me I had it, I became unfortunate… I am married, but because of this, I have no joy in my marriage,”* Lizzy said.

### Shock/Shame

A participant narrated that having been told she was HIV positive was a situation she least expected, and she found it utterly embarrassing.

“*I am human, so I feel shy about this disease. But, unfortunately, this disease is not curable; It lives with you for the rest of your life, and people think that when you get it, it is because you did something bad”* Joyce.

However, to some participants, they feel ashamed because of stigma and stereotyping HIV as resulting mainly from sexual contact and sexual narratives remain pervasive, as reflected in the narratives below:

*“I am surprised because it was about three years ago, and my mother was sick and needed blood. I am the only person out of her seven children who were able to donate for her, but since then, I didn’t have any affair with any lady, so I don’t know where I got it from. I came to this place because I was coming to marry a certain lady in this town, so the marriage committee of the church asked us to go for a check-up, and that was when I got to know I had it, so I called off the marriage because I didn’t want to worry her.”* Joyce.

*“Hmm, when I got to know about it, I became apprehensive because since my husband died, my children and I agreed that I was not going to have anything to do with any man, and I hadn’t had an affair with any man so in fact when I was told I got worried, it even affected my sleep, but I heard a voice that kept asking me not to worry so these days I don’t have problem with sleep”* Ama.

*“Truthfully, I fell sick for a long time, so finally, when I was told this was the disease. I felt apprehensive, so I kept thinking about where it came from. It wasn’t very easy for me. Sometimes I wake up in the middle of the night thinking about where it came from because I am not into men,”* Esther said.

## Discussion

The study explored the interpretation and reaction to HIV-positive status. Based on their perceptions and experiences, participants had varied understandings of their condition, which appeared to be influenced by several variables, including the disease’s chronicity and how family and society perceive an individual with HIV [46]. Participants interpreted HIV status as a challenge impacting all aspects of their social and economic life. Following analysis of these interpretations and the meaning ascribed to their HIV diagnosis, two major themes emerged: (1) anxiety regarding the impact of the disease. This was expressed in the following subthemes; stigma, fear, worry, shock, and shame. (2) Hopelessness, characterised by subthemes such as suicidal ideation, suicide plans and self-harm. In addition, the individuals’ perception and understanding of HIV influenced their psychosocial reactions, such as disappointment, loss, a different person, shame, unmentionable illness, stigma, and social and public rejection [46].

Narratives in this study suggest that the aftermath of an HIV-positive diagnosis is filled with immediate emotional reactions and potential long-term consequences [19]. Their immediate reactions ranged from shock, shame, fear and worry. The fear of HIV/AIDS relates to the fear of death, the most basic of fears [20]. These are consistent with previous findings [19]. Participants’ initial reactions suggested they were surprised and shocked to discover they were HIV positive. Some people reacted to the news of the HIV-positive diagnosis by denying it. However, denial may be normalising and an adaptive way to handle the shock associated with the HIV diagnosis [20]. In addition, participants expressed fear and worry regarding the public and societal reactions to their HIV status leading to self and anticipated social stigma [4]. Stigma is a phenomenon whereby an individual with an attribute such as HIV-positive who is deeply discredited by society is rejected due to the condition [4]. Stigma has been shown to act as a barrier to the prevention and treatment [21] because of feelings of shame, blame, guilt and social isolation [22]. Stigmatising attitudes, either experienced [23], perceived [24] or internalised [25], are important issues of concern for individuals with chronic health conditions such as HIV [26, 27]. Diagnosis with HIV can have adverse effects, including difficulties in personal relationships, disruption of vocational, professional and educational goals and, significantly, delay in seeking help [28, 29].

As a result of stigma, disclosure of HIV status was one of the most challenging issues that confronted participants in this study. Most participants were uncomfortable discussing HIV with their families and friends because of the associated stigma, discrimination, and rejection. As a result, participants reported they kept their diagnosis secret. They concealed their condition from people. They are uncomfortable disclosing their status to others because, in their view, a positive status means losing friends, removing their circle of friends, and causing perturbation in social relations. However, research suggests that HIV disclosure has negative and positive consequences [30, 31], resulting in complex decision-making. It is claimed that being open about this disease may simplify getting social support from significant others, such as families and friends, which appears to be a prerequisite for healthy coping, enhanced self-esteem, adherence, and other health-seeking behaviours [32]. Denial and disclosure of HIV status are part of the normalisation process, which can be positive and negative for the individual and their families [33]. Diagnosing a chronic disease such as HIV may lead to the individual adopting different strategies to cope with the condition, some of which may affect their health-seeking behaviours and families [33].

Studies on PLHIV have shown positive health outcomes. For example, a better immunological recovery is associated with disclosure [34]. In addition, disclosure may help to buffer HIV-related stress [35]. In contrast, HIV disclosure may also provoke fear of rejection and a breach of confidentiality [50], bearing the real risk of stigma and discrimination. Therefore, keeping one’s HIV status secret because of the many challenges of HIV stigma potentially limits people with HIV’s social support needs [51].

Furthermore, participants expressed hopelessness following the diagnosis. Although pharmacological agents have significantly improved the quality of life, HIV is still perceived as terminal and life-threatening. This interpretation often leads to pathological and maladaptive behaviours [52]. A similar study found that almost half of the participants reported hopelessness [53]. Consistent with other studies, there was an increased suicidal attempt among people with HIV [20]. Participants may consider suicide as a way out of the pain, misery, shame and grief [20]. Participants expressed that an HIV-positive status is synonymous with falling into despair [46]. They claimed that being diagnosed with an incurable disease like HIV is a taboo in society [46]. In contrast, other studies reported that a positive HIV diagnosis presents an opportunity for new life and goals, and the individuals become normalised [54]. The differences in the interpretation and reactions to HIV diagnosis suggest a need for more effective and culturally innovative interventions to support people receiving an HIV-positive diagnosis [46].

Many participants had suicidal ideations following diagnosis, and others in this study contemplated suicide but for the intervention’s of friends and health professionals. The outcomes of this study show the psychological and mental health challenges for persons diagnosed with HIV infection. This highlights how essential it is to assess suicidal ideation and provide timely interventions as part of mental health professionals’ responsibilities in HIV care. The frequency of suicide attempts and ideation in this study reflects other studies in Sub-Sahara Africa [55] and Western countries [49, 56]. However, research findings suggest that reported suicide rates are lower in Sub-Saharan Africa than in developed countries [57, 58], although suicidal deaths are more common in low-income countries [59]. These differences may reflect several factors, such as socio-cultural differences. All the participants in this study are religious, with a Christian majority. Both Christianity and Islam frown on suicide. Therefore religious and cultural components may have a place in the diagnosis of HIV and potential psychosocial reactions to receiving an HIV-positive diagnosis [2, 3]. For example, conservative religious and secular institutions disagree on abstinence, premarital and extramarital sex, contraception and homosexuality [2]. Some religious organisations insist on mandatory HIV testing before marriage [60], while others support the contrary [2]. Therefore, identifying with religious organisations and traditions promoting abstinence or monogamy could decrease the likelihood of participation in vulnerability-enhancing behaviours [3]. In contrast, religious teachings may be detrimental by discouraging certain protective measures, such as condom use, or denying young people safe sex education [61]. Advocates of religion as a protective factor also point out that religious affiliation fosters the creation of interpersonal networks that may enhance the diffusion of HIV-related information.

Furthermore, in addition to suicidal ideations, some participants reported having what appeared as suicide plans, although they did not disclose the nature and form of the suicide plan. This finding is consistent with earlier research [62, 63] and further suggests that individuals with active suicide plans will commit suicide in their lifetime [59].

It is also suggested that while assessing people with HIV, there is a need to consider the time interval following diagnosis. Although most people may perceive a diagnosis of HIV as a death sentence, it is claimed that this perception diminishes over time [64]. Research suggests that most suicide cases occur within the first four months after diagnosis [64]. Previous findings indicate that most participants who settled well after diagnosis benefited from psychosocial support from health professionals [48]. Therefore early mental health interventions must be made part of the package of interventions at diagnosis to support clients receiving an HIV diagnosis and thus prevent death.

### Limitations

1. The respondents were recruited through the HIV clinics where people receive HIV care and their medications. However, this method may miss out on others who do not access care at the HIV clinics due to stigma.
2. The study did not differentiate between participant who were recruited at the point of diagnosis but included others who could only recall the experiences.
3. Similar to research in Africa, most participants are females and Christians, which may require further research to explore the experience by demographic groups.

## Conclusion

The findings in this study revealed that people receive and respond differently to the diagnosis of HIV. The interpretations and reactions to such an incurable but treatable disease as HIV can have a debilitating effect on the individual’s psychological well-being if not well-managed. Suicidal ideation is one of the psychological consequences of receiving an HIV diagnosis reported in this study. In addition, the initial reaction to HIV-positive status in this LMIC context can impact care negatively by discouraging people from testing to know their HIV-positive status and, thus, a threat to achieving the global 95-95-95 goals. We recommend integrating mental healthcare with HIV care during and after testing to provide continuous support for individuals and families. This will facilitate early linkage to HIV care, improving overall outcomes for the infected individual and society.

## Data Availability

All information is included in the manuscript but can be made available upon reasonable request through the corresponding author.

## Abbreviations

HIV: Human Immunodeficiency Virus
PLHIV: People Living with HIV
ART: Antiretroviral Therapy
LMIC: Lower and Middle-Income countries
CCTH: Cape Coast Teaching Hospital
